# Supplementary material for: Comprehensive characterization of V(D)J recombination from long-read transcriptomic data with VDJcraft
Source: bioRxiv. 2026 Apr 5:2026.04.01.715879. Preprint. [Version 1] doi: 10.64898/2026.04.01.715879 (PMC13060111; doi:10.64898/2026.04.01.715879)
Supplement: Supplement 1 [file media-1.zip › VDJ_Supplementary_materials_v1_2026.pdf]

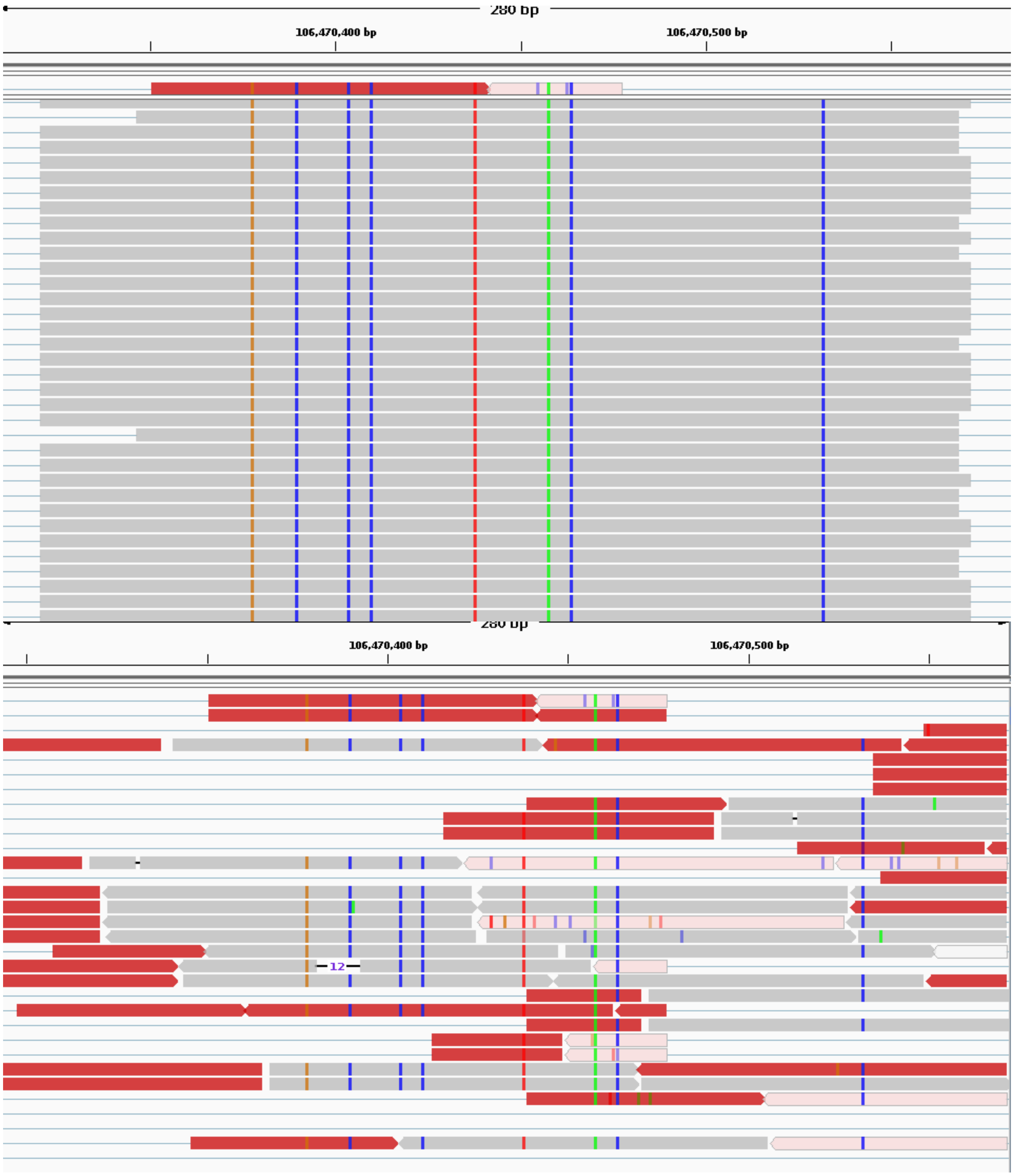

Supplementary Figure 1. Comparison of IGV view for IGHV3-43 gene between long reads and short reads. Upper panel shows long reads alignment in IGHV3-43 gene region, below panel shows short reads alignment in IGHV3-43 gene region.

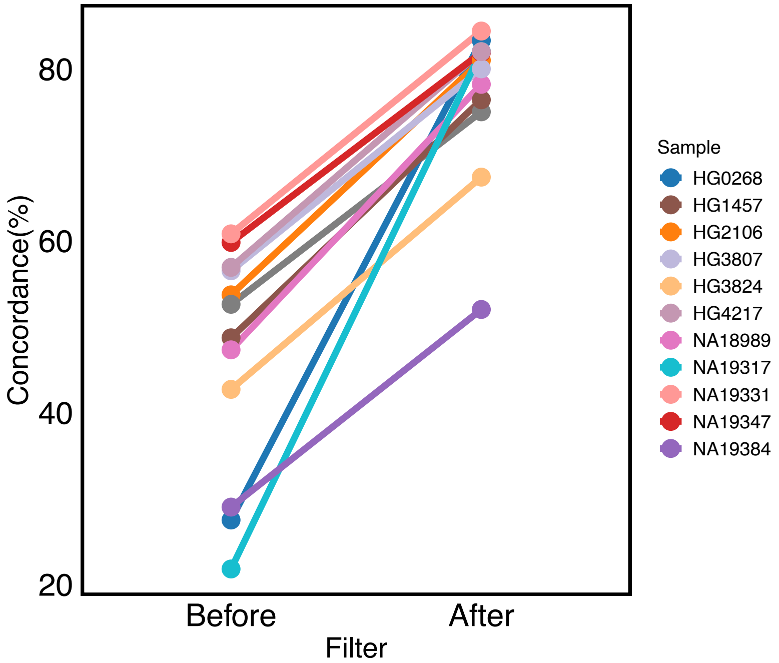

Supplementary Figure 2. Concordance of VJ recombination between long reads and short reads on HGSVC Samples VDJcraft and TRUST4. VJ recombination concordance between VDJcraft and TRUST4 increased markedly after filtering out low-supporting clonotypes compared to before filtering.

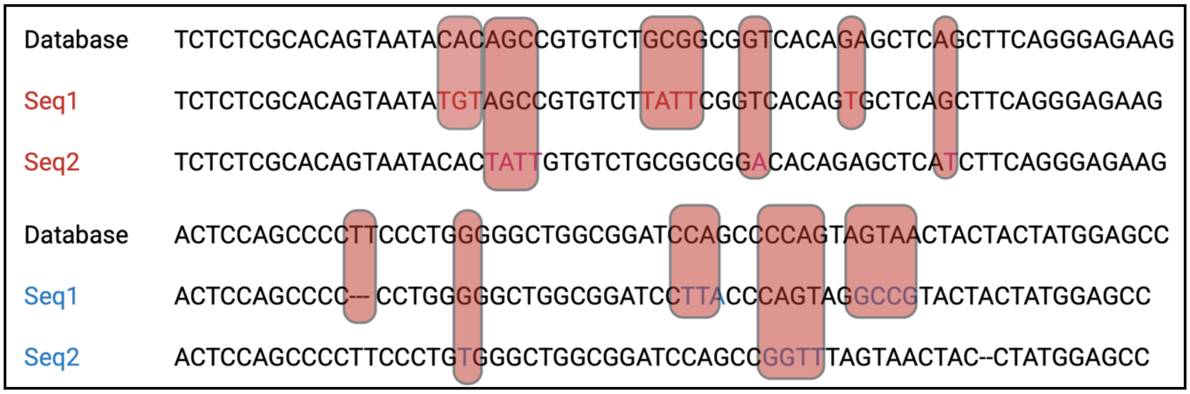

Supplementary Figure 3. Novel events identified by VDJcraft potentially induced by somatic hypermutation. Mismatched sequences are highlighted within the sequences, indicating regions where somatic hypermutation has occurred.

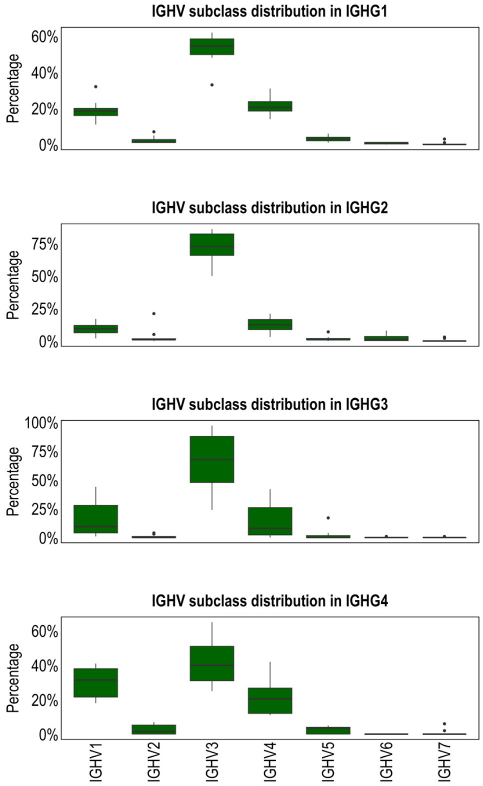

Supplementary Figure 4. IGHV subclass distribution of 10 FLAIRR samples using VDJcraft consistent with the results shown on previous literature. The trend of IGHV family usage across different IGHG subclasses detected by VDJcraft is consistent with previously reported findings[1].

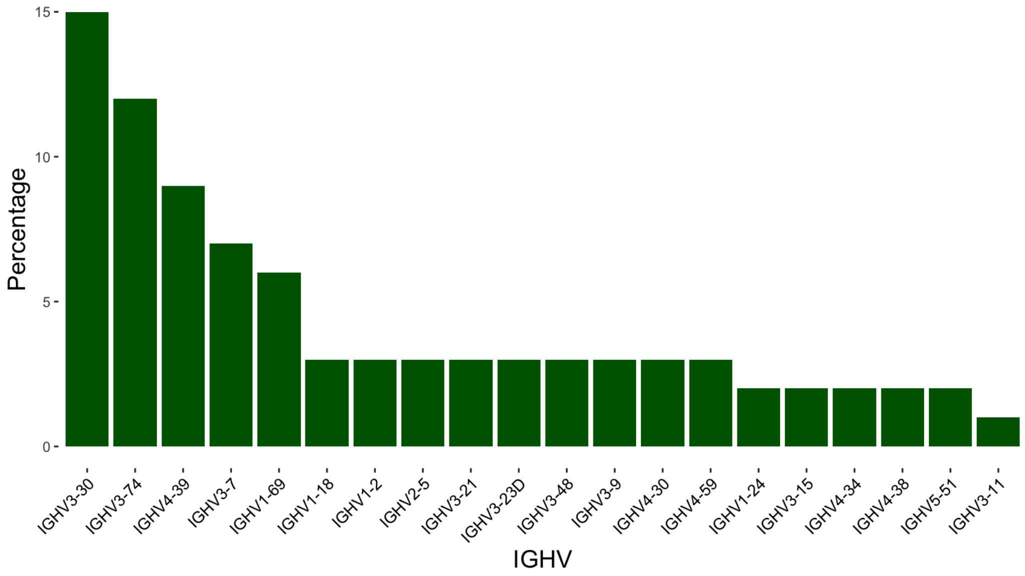

Supplementary Figure 5. Top 20 IGHV gene enrichment in FLAIRR1013 sample by percentage using VDJcraft consistent with the results shown on previous literature. IGHV3-30 exhibited the highest usage among IGHV genes in this sample, consistent with previously reported findings[1].

Supplementary Table 1. Comparison of VDJ detection on HGSVC sample (HG00268) by VDJcraft. TRUST4, and Lymanalyzer.

Measurement	VDJcraft	TRUST4	Lymanalyzer
**Outcome**	Success	Failed	Failed
**Time consuming**	4h 50min	-	50 hour of runtime or more
**Memory usage**	19.769G	(20 GB memory per CPU; 20 GB CPU per task)	8 GB memory per CPU; 5 GB CPU per task
**Error**	-	Out of memory	-
**Output**	Complete report	No output	No output

Supplementary Table 2. Comparison of top enriched CDR3 sequences identified by VDJcraft and TRUST4.

VDJ_clonotype	VDJcraft_CDR3nt	VDJcraft_CDR3aa	TRUST4_CDR3nt	TRUST4_CDR3aa
IGHV7-4-1_02 . IGHJ4_02	TGTGCGAGAGATGACCCTTACGAGCTTTTGACTGGTAATGACCTCTTT	CARDDPYELLTGNDLF	TGTGCGAGAGATGACCCTTACGAGCTTTTGACTGGTAATGACCTCTTTGACAAGTGG	CARDDPYELLTGNDLFDKW
IGLV1-47_02 . IGLJ3_02	GCAACATGGGATGACAGCCTGAGTGCTTGG	ATWDDSLSAW	TGTGCAACATGGGATGACAGCCTGAGTGCTTGGGTGTTC	CATWDDSLSAWVF
IGHV3-23_01 . IGHJ4_02	TGTGCGAAATGTTCCTGTAAAGCAGGGGCTGACCAACTTGACCACTGG	CAKCSCKAGADQLDHW	TGTGCGAAATGTTCCTGTAAAGCAGGGGCTGACCAACTTGACCACTGG	CAKCSCKAGADQLDHW
IGHV4-4_07 . IGHJ3_02	TGTGCGAGAGTCGGGGATAGTAGTGGTTATTACCCTGATGCTTT	CARVGDSSGYYPDAF	TGTGCGAGAGTCGGGGATAGTAGTGGTTATTACCCTGATGCTTTTGATATCTGG	CARVGDSSGYYPDAFDIW
IGKV1-39_01 . IGKJ1_01	TGTCAACAGAGTTACAGTACCCCTTGG	CQQSYSTPW	TGTCAACAGAGTTACAGTACCCCTTGGACGTTC	CQQSYSTPWTF
IGHV1-18_01 . IGHJ4_02	TGTGCGAGGGGAAAATATAGTAGTGGTTGGCCCGCTGACTACTGG	CARGKYSSGWPADYW	TGTGCGAGAACGGCGGTCTACTTCGGGGACTTTGACTCCTGG	CARGTDTVVVTAPIDYW
IGLV1-47_02 . IGLJ3_02	GCAACATGGGATGACAGCCTGAGTGCTTGG	ATWDDSLSAW	TGTGCAGCATGGGATGACAGCCTGAGTGGTCCAGGATGGGTGTTC	CAAWDDSLSGPGWVF
IGKV3-15_01 . IGKJ2_01	TGTCAGCAGTATAATAACTGGCCTCCGTACACTTT	CQQYNNWPPYTF	TGTCAGCAGTATAATAACTGGCCTCCGTACACTTTT	CQQYNNWPPYTF
IGHV4-4_07 . IGHJ4_02	TGTGCGAGGGGGGAGTATTACTATGATAGTAATGGGTATGACTACTGG	CARGEYYYDSNGYDYW	TGTGCGAGAACGGCGGTCTACTTCGGGGACTTTGACTCCTGG	CARTAVYFGDFDSW
IGLV2-14_01. IGLJ1_01	TGCAGCTCATATACAAGCAGCAGCACTTATGTCTTC	CSSYTSSSTYVF	TGCAGCTCATATACAAGCAGCAGCACTCGAGTCTTC	CSSYTSSSTRVF
IGHV3-11_01. IGHJ6_02	TGTGCGAGGACCCCGACTGGAAACAATTATGGTTCAGACTACTACTACTACGGTATGG	CARTPTGNNYGSDYYYYGMW	TGTGCGAGGACCCCGACTGGAAACAATTATGGTTCAGACTACTACTACTACGGTATGGACGTCTGG	CARTPTGNNYGSDYYYYGMDVW
IGHV4-34_10. IGHJ4_02	TGTGCGAGGGGAACAGACACTGTGGTGGTGACTGCTCCTATTGACTACTGG	CARGTDTVVVTAPIDYW	TGTGCGAGAGTGTTACGTCGAGTTACTACGATAGTAGTGGTTTTTGACTACTGG	CARVLRRVTTIVVVFDYW
IGHV3-64_07 . IGHJ3_02	TGCGAGAGCCCCCGCAGCCCTGAGGGCTGCTTT	CESPRSPEGCF	TGTGCGAGAGCCCCCGCAGCCCTGAGGGCTGCTTTTGATATCTGG	CARAPAALRAAFDIW
IGKV3-15_01. IGKJ3_01	TGTCAGCAGTATAATAACTGGCCTTTC	CQQYNNWPF	TGTCAGCAGTATAATAACTGGCCTTTCACTTTC	CQQYNNWPFTF
IGLV2-23_01 . IGLJ3_02	TGCTGCTTATATGCAGGTAACACTGATTGG	CCLYAGNTDW	TGCTGCTTATATGCAGGTAACACTGATTGGGTGTTC	CCLYAGNTDWVF
IGKV3-20_01. IGKJ2_01	TGTCAGCAGTATGGTGGCTCACCCCCGGGGTACACTTT	CQQYGGSPPGYTF	TGTCAGCAGTATGGTGGCTCACCCCCGGGGTACACTTTT	CQQYGGSPPGYTF
IGKV3-20_01. IGKJ4_01	TGTCAGCAGTATTCTGGCTCACAGAGGACTTT	CQQYSGSQRTF	TGTCAGCAGTATTCTGGCTCACAGAGGACTTTC	CQQYSGSQRTF
IGHV3-66 . IGHJ6_02	TGTGCGAGATTCGGCTATAGCAGCCCACCCAGACCGTTCTACTACTACGGTATGG	CARFGYSSPPRPFYYYGMW	TGTGCGAGATTCGGCTATAGCAGCCCACCCAGACCGTTCTACTACTACGGTATGGACGTCTGG	CARFGYSSPPRPFYYYGMDVW
IGHV3-48_01 . IGHJ4_02	TGTGCGAGCCCAAAGTCAGAGGGATCCAGGGATTACTTT	CASPKSEGSRDYF	TGTGCGAGCCCAAAGTCAGAGGGATCCAGGGATTACTTTGACTACTGG	CASPKSEGSRDYFDYW
IGLV1-44_01 . IGLJ1_01	TGTGCAGCGTGGGATGACAGCCTGAATGGCAATGTCTTC	CAAWDDSLNGNVF	TGTGCAGCGTGGGATGACAGCCTGAATGGCAATGTCTTC	CAAWDDSLNGNVF
IGKV3-20_01 . IGKJ1_01	TGTCAGCAGTATTCTGGCTCACAGAGGACTTT	CQQYSGSQRTF	TGTCAGCAGTATAGTAGCGCACCGTGGACGTTC	CQQYSGSQRTF
IGHV3-9_01 . IGHJ3_02	TGTGTGAAGGTAATACACAGTGCCATTGG	CVKVIHSAIW	TGTGTGAAGGTAATACACAGTGCCATTGGTGCTTTTGATATCTGG	CVKVIHSAIGAFDIW
IGLV2-14_01 . IGLJ3_02	TGCAGCTCATATACAAGCAGCAGCACTTATGTCTTC	CSSYTSSSSWVF	TGCAGCTCATATACAAGCAGCAGCTCTTGGGTGTTC	CSSYTSSSSWVF
IGKV3-15_01 . IGKJ4_01	TGTCAGCAGTATAATAAATGGCCGCTCACTTT	CQQYNKWPLTF	TGTCAGCAGTATAATAAATGGCCGCTCACTTTC	CQQYNKWPLTF
IGHV3-7_01 . IGHJ5_02	TGTGCGGGACTCAGCTACATGGCATTT	CAGLSYMAF	TGTGCGGGACTCAGCTACATGGCATTTGACCTCTGG	CAGLSYMAFDLW
IGLV2-23_02 . IGLJ3_02_IGLV2-11_01 IGLJ3_02)	TGCTGCTCATATGCAGGCAGCTACACTTTCGGGGTGTTC	CCSYAGSYTFGVF	TGCTGCTCATATGCACGTAGTAGCACCTGGGTGTTC	CCSYARSSTWVF
IGHV3-15_01 . IGHJ4_02	TGTACCACAGGGTATGGCCCTGACTACTGG	CTTGYGPDYW	TGTAGTACAGATGATTACGATATTTTGACTGGTTATTCCGATTACTGG	CSTDDYDILTGYSDYW
IGLV1-44_01 . IGLJ3_02	TGTGCAGCGTGGGATGACAGCCTGAATGGCAATGTCTTC	CAAWDDSLNGNVF	TGTGCAGCATTGGATGACAGCCTGAATGGCTGGGTGTTC	CAALDDSLNGWVF
IGLV3-21_03 . IGLJ3_02	TGTCAGGTGTGGGATAGTAGTTGGGGCGTCTTC	CQVWDSSWGVF	TGTCAGGTGTGGGATGGAGGTGTTGCCTGGGTGTTC	CQVWDGGVAWVF
IGHV1-2_02 . IGHJ5_02	TGTACGACATATCAGGACAACCAGTGGTTGCCCCCGTTC	CTTYQDNQWLPPF	TGTACGACATATCAGGACAACCAGTGGTTGCCCCCGTTCGACCCCTGG	CTTYQDNQWLPPFDPW
IGLV6-57_04. IGLJ3_02	TGTCAGTCTTATGATACCAGCAATCTCGTGTTC	CQSYDTSNLVF	TGTCAGTCTTATGATACCAGCAATCTCGTGTTC	CQSYDTSNLVF
IGKV1-5_01. IGKJ1_01	TGCCAACAGTATAATAGTTATTGG	CQQYNSYW	TGCCAACAGTATAATAGTTATTGGACGTTC	CQQYNSYWTF
IGHV3-9_01 . IGHJ6_02	TGTGTTAGAGCAGTGCCCGGAGAGGGCGGTATGG	CVRAVPGEGGMW	TGTGTTAGAGCAGTGCCCGGAGAGGGCGGTATGGACGTCTGG	CVRAVPGEGGMDVW
IGHV4-4_07 . IGHJ4_02	TGTGCGAGGGGGGAGTATTACTATGATAGTAATGGGTATGACTACTGG	CARGEYYYDSNGYDYW	TGTGCGAGGAGTCTTGATTGGCAGTATCCCTTTGACTTCTGG	CARSLDWQYPFDFW
IGLV1-44_01 . IGLJ3_02	TGTGCAGCATTGGATGACAGCCTGAATGGCTGG	CAALDDSLNGW	TGTGCAGCATGGGATGACAGTTTGAAGGGTTGGGTGTTC	CAAWDDSLKGWVF

IGHV1-2_02 . IGHJ5_02	TGTACGACATATCAGGACAACCAGTGGTTGCCCCCGTTC	CTTYQDNQWLPPF	TGTACGACATATCAGGACAACCAGTGGTTGCCCCCGTTCGACCCCTGG	CTTYQDNQWLPPFDPW
IGLV6-57_04 . IGLJ3_02	TGTCAGTCTTATGATAGCAGCGGCTGG	CQSYDSSGW	TGTCAGTCTTATGATACCAGCAATCTCGTGTTC	CQSYDTSNLVF
IGKV1-5_01 . IGKJ1_01	TGCCAACAGTATAATAGTTATTGG	CQQYNSYW	TGCCAACAGTATAATAGTTATTGGACGTTC	CQQYNSYWTF
IGHV3-9_01 . IGHJ6_02	TGTGTTAGAGCAGTGCCCGGAGAGGGCGGTATGG	CVRAVPGEGGMW	TGTGTTAGAGCAGTGCCCGGAGAGGGCGGTATGGACGTCTGG	CVRAVPGEGGMDVW
IGHV4-4_07 . IGHJ4_02	TGTGCGAGGGGGGAGTATTACTATGATAGTAATGGGTATGACTACTGG	CARGEYYYDSNGYDYW	TGTGCGAGGAGTCTTGATTGGCAGTATCCCTTTGACTTCTGG	CARSLDWQYPFDFW
IGLV1-44_01 . IGLJ3_02	TGTGCAGCATTGGATGACAGCCTGAATGGCTGG	CAALDDSLNGW	TGTGCAGCATGGGATGACAGTTTGAAGGGTTGGGTGTTC	CAAWDDSLKGWVF
IGKV3-15_01. IGKJ2_01	TGTCAGCAGTATAATAACTGGCCTCCGTACACTTT	CQQYNNWPPYTF	TGTCAGCAGTATAATAACTGGCCGTACACTTTT	CQQYNNWPYTF
IGHV3-7_01 . IGHJ4_02	TGTGCGAGAGATCAGTGGCGGATCTTT	CARDQWRIF	TGTGCGAGAGATCAGTGGCGGATCTTTGACTACTGG	CARDQWRIFDYW
IGLV3-9_01 . IGLJ1_01	TGTCAGGTGTGGGACGCCGGCACTGCAGGGTATGTCTTC	CQVWDAGTAGYVF	TGTCAGGTGTGGGACGCCGGCACTGCAGGGTATGTCTTC	CQVWDAGTAGYVF
IGKV1-33_01 . IGKJ5_01	TGTCAACAGTATGATAATCTCCCCCCGATCACCTTC	CQQYDNLPPITF	TGTCAACAGTATGATAATCTCCCCCCGATCACCTTC	CQQYDNLPPITF
IGHV2-5_02 . IGHJ4_02	TGTGCACACTTTGGCCAAGTCTACTTT	CAHFGQVYF	TGTGCACACTTTGGCCAAGTCTACTTTGACTACTGG	CAHFGQVYFDYW
IGHV3-66_01 . IGHJ6_02	TGTGCGAGAGATGGGTGGTTAGGGGCCGGGGGTTT	CARDGWLGAGGF	TGTGCGAGAGATGGGTGGTTAGGGGCCGGGGGTTTGGACGTCTGG	CARDGWLGAGGLDVW
IGHV4-61_12 . IGHJ5_02	TGTGCGAGACATTTACAGATTTTTGCCTGG	CARHLQIFAW	TGTGCGAGACATTTACAGATTTTTGCCTGGTTCGACCCCTGG	CARHLQIFAWFDPW
IGHV1-18_01. IGHJ4_02	TGTGCGAGAGGGAGAGACCCAGCTGCCGATCTTGACTTC	CARGRDPAADLDF	TGTGCGAGAGGGAGAGACCCAGCTGCCGATCTTGACTTCTGG	CARGRDPAADLDFW
IGLV6-57_04 . IGLJ3_02_IGHV3-23D_01 IGHJ1_01)	TGTGCGAACCAACAGTCCCAAAAACTACGGGAATACTTC	CANQQSQKLREYF	TGTGCGAACCAACAGTCCCAAAAACTACGGGAATACTTCCAGCACTGG	CANQQSQKLREYFQHW

Supplementary Table 3. Novel genes identified by VDJcraft potentially enrich IMGT database.

count	Gene_class	Novel_seq
8	IGHV1-18*	CTCTCGCACACTAATATAAGGCCGTGTCGTCAGATCTCAGGCTCCTCAGGTCCATGTAGAGTGTAGTCGCGGACGTGTCTCTGGTCAAGGTGACTCTGCCCTAGAACTGCTGTGTGTAGGTTGTGCTGCCA
11	IGHV1-18*	TGGCAGCACAACCTACACACAGCAGTTCTAGGGCAGAGTCACCTTGACCAGAGACACGTCCGCGACTACACTCTACATGGACCTGAGGAGCCTGAGATCTGACGACACGGCCTTATATTAGTGTGCGAGAG
4	IGHV1-69*	TCTCCCGCACAGTAATATACGGCCGTGTCTTCAGATGTCAGATTCGTTAGTTCCATGTAGACCATGTTTGTCTTCTTTTCCGCGGTCATTGTGACTCTGCCGTGGAATTTCTGTGCGTAGTGTGGAATATTAA<br>GTAGAGGGATGATCCTTCCCATCCACTCAAGTCCATGTCCTGGGGCCTGTCGCACCCAGCTGATACTATTACTGATGAGGGTGCCGCCAGAAGCCTTACAG
2	IGHV1-8*	CAGGTGCAGGTGTTGCATTCTGGGGCTGAGGTGAAGAAGCCTGGGGCCTCAGTGCAGGTCTCCTGGAAGGCTTCTGGATACACCTCCGTCACTTATGGTATCAACTGGGTGCGACAGGCCACTGGACACGG<br>GCTGCGACAGGCCACTGGACAAGGGCTTGAATGCATGGGATGGATGAACCCTAACAGTGGTGGCACAGGCTATGCACAGATTTTCCAGGGCAGAGTCACCATG
2	IGHV1-8*	TCTCGCACAATAATACGCGGCCGTGTCTTCAAATCTCAGGCTGCTCATCGCCATGCAGCCTGTGTTTATGGAGGTGTTCCTGGCCATGGTGACTCTGCCCTGGAAAATCTGTGCATAGCCTGTGCCACCACT<br>GTTAGGGTTCATCCATCCCATGCATTCAAGCCCTTGTCCAGTGGCCTGTCGCAGCCCGTGTCCAGTGGCCTGTCGCACCCAGTTGATACCATAAGTGACGGA
2	IGHV3-15*	GTGGTTCAAAAAGACACGCCTGTGTCCTCGGTTTTCAGGCTGTTCATTTCCAAACACAGCGGAGTTTTCGGTTAATCTCTTCAGACGGTGAATCAGGCTTTCACGGGCACAACGTAGTCCGGTGCCCCAACAT<br>CACCTTTTGGTTTTAAGACGGCCAACCCACTCCAGCCCCCTCCCTGCAGCCTGGCGGACCCACCTCATCC--------------GTGAATCCAGAGTCTAC
2	IGHV3-23D_*	GAGGTACAATTGTTGGATTCTGGGGGAGGCTTGGTACAGCCCTGGGGGGTCCCTGAGACTCTCCTGTTCAGCCGCTGGATTCACGTTTACCAACTTTGCCATGACCTGGGTC-GCCAGGCTCC-GGGAGGGGG<br>CTCGAGTGGGTCTCAACTTTATTCCTAGTGACGATAGAGTAAACTACGATAGAACATACTACACAGACTCCGTGAAGGGCCGGTTCACCATCTCCAGAGAC
2	IGHV3-33_*	CTCTTGCACATTAATACACAGCCGTGTCCCCGGCTCTCAGGCTGTTCATTTGAAGATACAAGGAGTTCTTGGCATTTTCTCTGGAGATGGTGAATCGGCCCTTCACGGAGCCTGGATCCTCTATGTCACCAG<br>CA-CTT-CCA-ATAGTTGAGACCCATTCCAGACCTTTTCCTGTCGCTTGGCGGACCCAGTGCATGTCGTAGCTACTGAAGGTGAATCCAGAGGCTGCACAGG
8	IGHV3-48_*	GACGTGCAGCTGGTGGAGTCGGGGGGACACTTGGTCCAGCCgggggggTCCCTGAGGCTCTCCTGTGCAGCCTCTGGATTCAGTTTTAGTAGTTTTTGGATGAGTTGGGTCCGCCAGGCTCCAGGGAAGG<br>GCCTCCAATGGGTGGCCAACA-TAAACCAAGATGGAACTGAGGA-ACACTATGTGGACTCTGTGCAGGGCCGCTTCACCATCTCCAGAGACAACGCCAGGAATT
7	IGHV3-48_*	TCTTTCGCACAGTGATAAACAGCCGTGTCCTCGGCTCTCAGGCTGAACAGTTGCAGAAACACTGAATTCCTGGCGTTGTCTCTGGAGATGGTGAAGCGGCCCTGCACAGAGTCCACATAGTGT-TCCTC<br>AGTTCCATCTTGGTTTA-TGTTGGCCACCCATTGGAGGCCCTTCCCTGGAGCCTGGCGGACCCAACTCATCCAAAAACTACTAAAACTGAATCCAGAGGCTGCAC
2	IGHV3-48_*	GAGGTGCAGGTGGTGGAGTCTGGGGAAGAGTTGCTACAGTTggggggggTCCCTGAGACTCTCCTGTGCAGCCTCTGGATTCACCCTCAGGCGAAACTGCATGAACTGAGTCCGCCAGGCTCCAGGGCAG<br>GGGCTGGAGTGGGTTTCAAACACTAGTGGAAGTTTTAATTTTAAAACCACATCTTAGGCAGACTCTCTGAAGGGCCGGTTCCCCATTTCCAGAGACAACGCCAA
57	IGHV3-48_04	GAGGTGCAGGTGGTGGAGTCTGGGGAAGAGTTGCTACAGTTgggggggTCCCTGAGACTCTCCTGTGCAGCCTCTGGATTCACCCTCAGGCGAAACTGCATGAACTGAGTCCGCCAGGCTCCAGGGCAGG<br>GGCTGGAGTGGGTTTCAAACACTAGTGGAAGTTTTAATTTTAAAACCACATCTTAGGCAGACTCTCTGAAGGGCCGGTTCCCCATTTCCAGAGACAACGCCAAC

28	IGHV3-48_*	TAAGACGCAGACGCGTCTCCGGCTCTCAGGCTGCTCATTCGCAGAAAGAGTGAGTTGTTGGCGTTGTCTCTGGAAATGGGGAACCGGCCCTTCAGAGAGTCTGC<br>CTAAGATGTGGTTTTAAAATTAAA<br>ACTTCCACTAGTGTTTGAAACCCACTCCAGCCCCTGCCCTGGAGCCTGGCGGACTCAGTTCATGCAGTTTCGCCTGAGGGTGAATCCAGAGGCTGCACAGGAGA<br>GT
3	IGHV3-48_*	TAAGACGCAGACGCGTCTCCGGCTCTCAGGCTGCTCATTCGCAGAAAGAGTGAGTTGTTGGCGTTGTCTCTGGAAATGGGGAACCGGCCCTTCAGAGAGTCTGC<br>CTAAGATGTGGTTTTAAAATTAAAA<br>CTTCCACTAGTGTTTGAAACCCACTCCAGCCCCTGCCCTGGAGCCTGGCGGACTCAGTTCATGCAGTTTCGCCTGAGGGTGAATCCAGAGGCTGCACAGGAGAG<br>T
2	IGHV3-72_*	ACAGTAATACACGGCTGTGTCCTCGGTTTTCAGGCTGTTCATTTGCAGATACAGCGTGTTTTTGAATCATCTCTTGAGATGGTGAATCTGCCTTTCACGGGTGCA<br>GCGTAGTCTGTTGTCCCACCATCAGTTTTGCTTTTAATACGGCC-A---AC-CCA----C-T-CC---AG-CT--------CA-TCCA-G--G-<br>CGTTACTGAAAGTGAATCCAGAGGCTGCACAGGA
2	IGHV3-72_*	GAGGTGCAGCTGGTGGAGTCTGGGGGAGGCTTGGTAAAGCCTGGGGGGTCCCTTAGACTCTCCTGTGCAGCCTCTGGATTCACTTTCAGTAACG-C--C-TGGA-<br>TG--------AG-CT---GG-A-G----TGG-GT---T-GG<br>CCGTATTAAAAGCAAAACTGATGGTGGGACAACAGACTACGCTGCACCCGTGAAAGGCAGATTCACCATCTCAAGAGATGATTCAAAA
2	IGHV3-OR16-9_*	GAGGTGCAGTTGGGGGAGTCTGGGGGAGGCCTGGCACAGCCTGGAGGGTCCCTGAGATTCTCCTGTCCCTCCTCTCGATTCACACTT-<br>AATAATTTTAACATGGAG-TGGGTCCGCCAGGCTCCAGGGAAGG<br>GACTGGAGTGGGTTTCAGAGATTAGTG--AATAGC----A-AG-AACTTCGCAGACTCTGTGAAGGGCCGATTCAGCATCTCCAGAGACAACGCCAGGAGCT
4	IGHV4-28_*	CAGGTGCAGCTACAGGAGTCGGGCCCAGGACTGGCGAAGCCTTCGGAGACTTTGTCCCTCACCTGCAGTGTCTCTGGTGGCTCCAT--G-<br>AGTAATTACTTCTGGAGCTGGATCCGGCAGCCCGCC-GGAAGG<br>GACTGGAGTGGCTTGGGCGTATGTATACCAATGGGAGGACCGACTACAACCCCTCCCTCAAGAGTCGACTCACCATGTCAATAGACATGTCTAAGAACACC
2	IGHV4-28_*	CAGGTGCAGCTACAGGAGTCGGGCCCAGGACTGGCGAAGCCTTCGGAGACTTTGTCCCTCACCTGCAGTGTCTCTGGTGGCTCCAT--G-<br>AGTAATTACTTCTGGAGCTGGATCCGGCAGCCCGCC-GGAAGG<br>GACTGGAGTGGCTTGGGCGTATGTATACCAATGGGAGGACCGACTACAACCCCTCCCTCAAGAGTCGACTCACCATGTCAATAGACATGTCTAAGAACACC
2	IGHV4-34_*	CAGGTGCAGCTACAACAGTGGGGCGCAGGCCTGTTGAGGCCCTCGGAGACCCTGTCCCTCACCTGCGCTCTGTCTGGTGGGTCCTTCAGTGGTTACTACTGGAC<br>CTGGATTCGCCAGTCCCCAGGGAGG<br>GGACTGGAGTGGATTGGTGAAATCAATCATAGTGGGATTGGTGAAATCAATCATAGTGGGAGTACCAACTACAACCCGTCCCTCAAGAGTCGAGTCACCATAT<br>CA
13	IGHV4-39_*	CAACTGCAGCTGCAGGAGTCGGGCCCAGGACTGGTGAAGCCTTCGGAGACCCTGTCCCTCACCTGCGCTGTTGCTGGTGGCTCCATTAGCAGTGGCAGTTAGTT<br>CCGGGGCTGGGTCCGCCAGCCCCCAGGG<br>AAGGGGCTGGTGTGCATTGAGAATATGTACTAAACTGATATCACCCACTACAACCCGTCCCTCAGGAGTCGAGTCACCATATCCCAGTCACCATATCCGTAG
2	IGHV4-39_*	CTCGCGCACTAGTAAACAGCCGCGCCTGCGGCGGTCAGCGAGATCAGCGTCAGGGAGAGCTCGTTCTGCGTCGTGTCCTCGCACACGGTGACTCGACAC---A-<br>GGA-----TGGAGGAGGATG-TCCCACTATAG<br>GAGAGACTCTCAATCCACCCCAGCGCCATCCCTGGGGGCTGGCGGTTCCAGCCCCAGGACTCAATATTACATCTGATGGGGCCACCAGAGACAGTGCA
8	IGHV4-39_*	TGTCTCGCACAGTTAGAAAGAGCCGTGTCTGCGGCGGTCACAGACCTCAGCCTCAGGGAGAACCAGTTCTTGGACGGGTCTACGGATATGGTGACTGGGATATG<br>GTGACTCGACTCCTGAGGGACGGGTTGT<br>AGTGGGTGATATCAGTTTAGTACATATTCTCAATGCACACCAGCCCCTTCCCTGGGGGCTGGCGGACCCAGCCCCGGAACTAACTGCCACTGCTAATGGAGC
2	IGHV4-39_*	CCCTGTCCCTCACCTGCACTGTTTTTGGTGGCCCCATCGCCACTGACGA-<br>TCACCACTGGGGCTGGATCCGCCAGTCCCCGGGGAAGGGACTGGAGTGGATTTTGAGTGTCTATTATCGTGGGAGCGCCTATCG<br>TGGGAACACCTACTGCAATCCGTCCCTCAAGGGACGAGTCGCCGTGTCCGTAGAGGCGTCCAGGAACAACTTCTCCCTGAAACTGAGCTCTGTGACCGCC
6	IGHV4-39_*	TCTCGCACAGTAATAGACAGCCGTGTCTGCGGCGGTCACAGAGCTCAGTTTCAGGGAGAAGTTGTTCCTGGACGCCTCTACGGACACGGCGACTCGTCCCTTGA<br>GGGACGGATTGCAGTAGGTGTTCCCACGA<br>TAGGCGCTCCCACGATAATAGACACTCAAAATCCACTCCAGTCCCTTCCCCGGGGACTGGCGGATCCAGCCCCAGTGGTGATCGTC-AGTGGCGATGGGGC
12	IGHV4-39_*	TGCTGCTGCAGGAGTCGGGCCCAGGACTGGTGAAGCCTTCGGAGACCCTGTCCCTCACCTGCACTGTTTTTGGTGGCCCCATCGCCACT-<br>GACGATCACCACTGGGGCTGGATCCGCCAGTCCCCGGGGAAGGG<br>ACTGGAGTGGATTTTGAGTGTCTATTATCGTGGGAGCGCCTATCGTGGGAACACCTACTGCAATCCGTCCCTCAAGGGACGAGTCGCC<br>GTGTCCGTAGAG
2	IGHV4-OR15-8_*	CAGGTGCAGTTGCAGGAGTCGGGCCCAGGGCTGGTGAAGGCTTCGGAGACCCTGTCCCTCATGTGCACTGTCTCTGGTGACCTCACCAACACTTAT-<br>ACTACTGGAACTGGATCCGGCAG-GCCCGGGGAGG<br>GGACTCGAATGGATTGGGTTTGTC-ACCAACAGTGGGAGAAGCAACTCCAACCCCT-CCTCAAGAGTCGAGTCACCATATCAATAGACACATCCAAGAACCA
2	IGHV4-OR15-8_*	TCTCTCGCACAGTAATAAACGGCCGTGTCCGCAGAGGTCACAGAGGTCAGCCTCAGGGAGAACTGGTTCTTGGATGTGTCTATTGATATGGTGACTCGACTCTT<br>GAAGGAGGGGTTGGAGTTGCTTCTCCCACT<br>GTTGGT-GACAAACCCAATCCATTCGAGTCCCCTCCCCGGGGCCTGCCGGATCCAGTTCCAGTAGTAAGTGTTGGTGA-GGT--CACCAGAGACAGTGCA
4	IGLV2-23_*	CAGTCTGCCCTGACTCAGCCTGCCTCCGTGTCTGGGTTTCCTGGACAGTCGAACACCATCCCCTGCACTGGAGCCAGCAGGGATGTTGGGGGATATTAATTTCTC<br>GCCTGGCTGCAACACCACCCAGACAAGGT<br>CCCCAAACTCACGATTGATGACGTCTCTAAGCGGCCCGCAGATACTACTACCTGCTTTTCTGGCTCCAAGTCTGGCAACGCGCCCTCCCTGACAGTCGCT
9	IGLV2-23_*	GCAGAAG-AA<br>AATCAGCCTCGTCCCCAGCCGGGAGCCCAGCGACTGTCAGGGAGGGCGCGTTGCCAGACTTGGAGCCAGAAAAGCAGGTAGTAGTATCTGCGGGCCGCTTAGA<br>GACGTCATCAATCGTGAGTTTGGGGACCTT<br>GTCTGGGTGGTGTTGCAGCCAGGCGAGAAATTAATATCCCCCAACATCCCTGCTGGCTCCAGTGCAGGGGATGGTGTTCGACTGTCCAGG
2	IGLV3-16_*	TCCTATGAGCTGACACAGCCACCCTCGGTGTCAGTGTCGCTAGGACAGATGGCCAGGATCGCCTGCTCCGGGGGAACCATCGTCAGAAAAATGTGATTCTTTCT<br>TGTACCAG-A-AATT-AGGCCAGTTCCCTGTG<br>CTGGT----------------GAGAGGCCCTCAGGGATCCCTGACCGATTCTCTGGCTCCACTTCAGGGACAATAGTCACATTGACCAGCAATGGAGT

* Indicate events belong to this gene subclass but unknown annotation.

Reference

1 Ford, E. E. *et al.* FLAIRR-Seq: A Method for Single-Molecule Resolution of Near Full-Length Antibody H Chain Repertoires. *J Immunol* **210**, 1607-1619 (2023). https://doi.org/10.4049/jimmunol.2200825
